# Determinant factors for first-line treatment choice and effectiveness in pediatric eosinophilic esophagitis: an analysis of the EUREOS EoE CONNECT registry

**DOI:** 10.1007/s00431-024-05618-z

**Published:** 2024-05-31

**Authors:** Pilar Navarro, Sara Feo‐Ortega, Sergio Casabona‐Francés, Carolina Gutiérrez‐Junquera, Edoardo V. Savarino, Edurne Amorena, Sonia Fernández‐Fernández, Isabel Pérez‐Martínez, Salvatore Oliva, Jesús Barrio, Maria Lluisa Masiques‐Mas, Antonio Guardiola‐Arévalo, Danila Guagnozzi, Francesca Racca, Elena Betoré, Martina Votto, Alba Rodríguez-Sánchez, Mónica Llorente Barrio, Leonardo Blas‐Jhon, Carlos Teruel Sánchez‐Vegazo, Natalia García-Morales, Anne Lund Krarup, Raffaella Dainese, Verónica Martín‐Dominguez, Alejandro García-Díaz, Daria Maniero, Cecilio Santander, Ángel Arias, Emilio J. Laserna‐Mendieta, Alfredo J. Lucendo

**Affiliations:** 1 Department of Gastroenterology, Hospital General de Tomelloso, Vereda de Socuéllamos s/n, 13700 Tomelloso, Spain; 2 Instituto de Investigación Sanitaria de Castilla‐La Mancha (IDISCAM), Toledo, Spain; 3 Instituto de Investigación Sanitaria La Princesa, Madrid, Spain; 4 Centro de Investigación Biomédica en Red de Enfermedades Hepáticas y Digestivas, CIBERehd, Madrid, Spain; 5 Department of Pediatrics, Hospital General de Tomelloso, Tomelloso, Spain; 6 Department of Gastroenterology, Hospital Universitario de La Princesa, Madrid, Spain; 7 Pediatric Gastroenterology Unit, Hospital Universitario Puerta de Hierro, Majadahonda, Spain; 8 Universidad Autónoma de Madrid, Madrid, Spain; 9 Department of Surgery, Oncology and Gastroenterology, University of Padua, Padua, Italy; 10 Gastroenterology Unit, Azienza Ospedaliera di Padova, Padua, Italy; 11 Department of Gastroenterology, Hospital Universitario de Navarra, Pamplona, Spain; 12 Department of Pediatric Gastroenterology, Hospital Universitario Severo Ochoa, Leganes, Spain; 13 Department of Gastroenterology, Hospital Universitario Central de Asturias, Oviedo, Spain; 14 Diet, Microbiota and Health Group, Instituto de Investigación Sanitaria del Principado de Asturias (ISPA), Oviedo, Spain; 15 Pediatric Gastroenterology and Liver Unit, Maternal and Child Health Department, Sapienza‐University of Rome, Rome, Italy; 16 Department of Gastroenterology, Hospital Universitario Rio Hortega, Valladolid, Spain; 17 Department of Pediatric Gastroenterology, Hospital de Granollers, Granollers, Spain; 18 Department of Gastroenterology, Hospital Universitario de Fuenlabrada, Fuenlabrada, Spain; 19 Department of Gastroenterology, Hospital Universitario Vall d’Hebrón, Barcelona, Spain; 20 Personalized Medicine, Asthma and Allergy Clinic, IRCCS Humanitas Research Hospital, Rozzano - Milan, Italy; 21 Department of Gastroenterology, Hospital Universitario Miguel Servet, Zaragoza, Spain; 22 Pediatric Unit, Department of Clinical, Surgical, Diagnostic and Pediatric Sciences, University of Pavia; and Pediatric Clinic, Fondazione IRCCS Policlinico San Matteo, Pavia, Italy; 23 Department of Gastroenterology, Hospital Universitario Santa Lucía, Cartagena, Spain; 24 Department of Gastroenterology, Hospital de Santa Bárbara, Soria, Spain; 25 Department of Gastroenterology, Fundación Jiménez Díaz, Madrid, Spain; 26 Department of Gastroenterology, Hospital Universitario Ramón y Cajal, Madrid, Spain; 27 Department of Gastroenterology, Hospital Álvaro Cunqueiro, Vigo, Spain; 28 Department of Emergency Medicine and Trauma Center, Aalborg University Hospital, Aalborg, Denmark; 29 Department of Clinical Medicine, Aalborg University, Aalborg, Denmark; 30 Department of Gastroenterology, Centre Hospitalier d’Antibes Juan‐les Pins, Antibes, France; 31 Department of Medicine, Universidad Autónoma de Madrid, Madrid, Spain; 32 Research Unit, Hospital General Mancha‐Centro, Alcázar de San Juan, Spain

**Keywords:** Adolescent, Eosinophilic esophagitis, Pediatrics, Proton pump inhibitors, Swallowed topical corticosteroids, Dietary intervention, Therapeutic use, Fibrosis

## Abstract

This study compared short-term effectiveness of proton pump inhibitors (PPI), swallowed topical corticosteroids (STC), and dietary therapies in reversing clinical and histological features in pediatric patients with eosinophilic esophagitits (EoE). Determinants for treatment choice and PPI therapy effectiveness were also assessed.  A cross-sectional study analysis of patients under 18 years old recruited onto the multicenter EoE CONNECT registry was performed. Clinico-histological response was defined as symptomatic improvement plus a peak eosinophil count below 15 per high-power field after treatment. Effectiveness of first-line options used in monotherapy was compared. Overall, 393 patients (64% adolescents) receiving PPI, STC, or dietary monotherapy to induce EoE remission were identified. PPI was the preferred option (71.5%), despite STC providing the highest clinico-histological response rates (66%) compared to PPI (44%) and diet (42%). Logistic regression identified fibrotic features and recruitment at Italian sites independently associated to first-line STC treatment; age under 12 associated to dietary therapy over other options. Analysis of 262 patients in whom PPI effectiveness was evaluated after median (IQR) 96 (70–145) days showed that this effectiveness was significantly associated with management at pediatric facilities and use of high PPI doses. Among PPI responders, decrease in rings and structures in endoscopy from baseline was documented, with EREFS fibrotic subscore for rings also decreasing among responders (0.27 ± 0.63 *vs.* 0.05 ± 0.22, *p* < 0.001).

*Conclusion*: Initial therapy choice for EoE depends on endoscopic phenotype, patient’s age, and patients’ origin. High PPI doses and treatment in pediatric facilities significantly determined effectiveness, and reversed fibrotic endoscopic features among responders.

**Table:** 

**What is Known:** *• Proton pump inhibitors are widely used to induce and maintain remission in EoE in real practice, despite other first-line alternative therapies possibly providing higher effectiveness.*	
**What is New:** *• Proton pump inhibitors represent up to two-thirds of first-line monotherapies used to induce EoE remission in pediatric and adolescent patients with EoE. The choice of STC as first-line treatment for EoE was significantly associated with fibrotic features at baseline endoscopy and recruitment in Italian centers; age less than 12 years was associated with dietary therapy.* *• PPI effectiveness was found to be determined by use of high doses, attendance at pediatric facilities, presenting inflammatory instead of fibrotic or mixed phenotypes, and younger age. Among responders, PPI therapy reversed both inflammatory and fibrotic features of EoE after short-term treatment.*

**Supplementary Information:**

The online version contains supplementary material available at 10.1007/s00431-024-05618-z.

## Introduction

Eosinophilic esophagitis (EoE) is a chronic, immune‐mediated inflammatory disease that is characterized by esophageal dysfunction and transmural infiltration by eosinophils restricted to the esophagus [1, 2], and generally triggered by exposure to dietary antigens [3]. EoE presents a chronic natural course in the vast majority of cases, and appears to be progressive, with long-standing eosinophilic inflammation leading to esophageal remodeling with stricture formation and functional damage [4–6]. Dysphagia and food impaction are the most characteristic symptoms in adults and adolescents, while symptoms reminding gastroesophageal reflux disease and feeding disturbances predominates in children [1, 7, 8].

The global prevalence of EoE currently exceed 60 cases per 100,000 inhabitants [9]; with a steady rise in EoE incidence and prevalence rates having been observed over time in patients of all ages [10]. Peak ages for EoE diagnosis are pediatric [11] and the third to fourth decades in life in adulthood [12]. Despite been a single disease across the age range, some symptoms are considered characteristic of pediatric EoE [13]; however, there is little research comparing the effectiveness of different treatment options for different age groups [7].

Anti-inflammatory first-line therapies for EoE consist of swallowed topical corticosteroids (STC), dietary modifications aimed at avoiding exposure to trigger foods, and proton pump inhibitors (PPIs) [14, 15]; an interleukine-4 receptor monoclonal antibody has recently been added to anti-inflammatory therapies [16]. Esophageal dilation should be considered a solution to fibrotic sequelae of EoE [17]. Despite its limited effectiveness of only 50% in inducing histological remission [18], off-label PPI therapy is currently the preferred initial therapy for EoE in patients of all ages and in most clinical settings [19–24]. This is likely due to drug availability, convenience, cost, and safety profile. However, the reasons determining treatment choice in pediatric EoE have not yet been analyzed, and determinants for PPI effectiveness in younger patients have been only limitedly assessed [25, 26].

In this study, we compare the short-term effectiveness of PPI, SCT, and dietary therapies in reversing clinical, histological, and, whenever possible, endoscopic features of EoE in pediatric patients, and aim to identify determinants for treatment choice and aspects underlying PPI therapy effectiveness in a large, collaborative, multicenter European registry.

## Methods

### Study design and data collection

The “European Registry of Clinical, Environmental and Genetic Determinants in Eosinophilic Esophagitis” (EoE CONNECT) is an international, multicenter, non-interventional registry initially promoted by United European Gastroenterology and currently supported by EUREOS, the European Consortium for Eosinophilic Diseases of the Gastrointestinal Tract (www.eureos.online).

A cross-sectional analysis of EoE CONNECT was performed [27]; patients aged younger than 18 years old and diagnosed with EoE based on evidence-based [1] and AGREE consensus criteria [2], and who received PPIs, STC, or any dietary interventions as first-line monotherapy to induce EoE remission were identified. Prospective clinical and demographic data from EoE patients had been imputed onto the registry by practitioners during face-to-face clinical appointments.

Variables collected for this study were sex, age, time of diagnosis, country of recruitment, type of hospital facility (pediatric or adult), EoE phenotype, histology findings, endoscopic features, and treatment response. Definitions, detailed study protocols and operational procedures of EoE CONNECT have been published elsewhere [27]. EoE CONNECT runs in accordance with the Declaration of Helsinki and has been approved by La Princesa University Hospital Research Ethics Committee (as central Committee) and Ethics Committees at all participating sites; assent and written informed consent to participate in the EoE CONNECT project was obtained, respectively, from all patients and their legal guardians.

### Main definitions

Histological remission was defined as an eosinophil peak count of < 5 eosinophils/high power field (eos/hpf) at all esophageal levels after therapy; histological response was considered as a peak count between 5 and 14 eos/hpf. Symptomatic improvement in adolescents was assessed by changes in the Dysphagia Symptom Score (DSS) [27], reported by patients or by clinicians’ perception. For younger children, any subjective improvement in symptoms reported by either children or parents was considered as a clinical response. In addition, clinicians semi-quantitatively expressed changes in symptoms from treatment initiation as a clinical response or no response.

Clinico-histological response was defined as the simultaneous combination of symptomatic improvement and histological response (peak eosinophil count below 15  eos/hpf) in the same patient after therapy; clinico-histological remission was defined as any symptomatic improvement together with < 5 eos/hpf after therapy.

Endoscopic features in the esophagus were graded by the presence and severity of edema, rings, exudates, furrows, and stricture(s) in accordance with the EREFS grading scoring system [28]. Rings and strictures were classified as fibrotic features, while edema, furrows, and exudates were defined as inflammatory ones [29].

Standard doses of PPI included omeprazole 20 mg, pantoprazole 40 mg, esomeprazole 20 mg, lansoprazole 30 mg, and rabeprazole 20 mg daily. Double doses or higher were considered high-dose PPI, and a low dose was defined when PPIs were given at standard doses or below [30–33]. For children under 12 years, the dosage in mg/kg was estimated by dividing the daily PPI dose by expected weight for age and sex in the pediatric population [34, 35].

### Statistical analysis

Means and standard deviations (or medians and interquartile ranges for non-normal variables) were reported for continuous variables, and proportions for categorical data. Frequency tables were generated for first-line treatment choice and effectiveness (clinico-histological response). Contingency tables to assess demographic and clinical factors influencing treatment choice and effectiveness were produced and analyzed by chi-square or Fisher exact (univariate) tests.

A binary logistic multivariate regression analysis was performed to assess demographical and clinical factors influencing treatment response rates of PPI treatment identified in univariate analyses. Additionally, a multinomial logistic regression was performed to assess demographic and clinical factors influencing first-line treatment choice identified in univariate analyses. Odds ratio (OR) and their 95% confidence interval (95%CI) were reported for those variables reaching statistical significance in each model.

Changes from baseline in EREFS score and sub-scores induced by PPI therapy were compared by the McNemar test (qualitative variables) and paired *t*-test (quantitative variables).

All analyses were carried out using PASW v18.0 statistical analysis software (SPSS Inc, Chicago, ILL, USA). A p-value < 0.05 was considered significant.

## Results

### Demographic and clinical characteristics of pediatric patients with EoE included in the study

On the search date, August 25th, 2023, 416 patients registered in EoE CONNECT and recruited across 29 study sites had received their first treatment for EoE when under 18 years old. PPIs were the main first-line treatment option (67.5%), while combination treatments were rare (5.3%) (Suppl Table 1). The main demographic and clinical characteristics of the 393 EoE patients treated with the three most common options (PPIs, STCs, and dietary interventions) in monotherapy were summarized in Table 1. They were mainly male (80%), adolescents between 12 and 18 years old (64%), with an inflammatory phenotype (90%) lacking endoscopic features of fibrosis (68%), and recruited in adult facilities (78%) at Spanish sites (84%).

**Table 1:** Demographic and clinical characteristics of pediatric patients with eosinophilic esophagitis (EoE), and treated with proton-pump inhibitors, swallowed topical corticosteroids or dietary interventions in monotherapy as first-line treatment

Number of patients, *n* (%) Children (0–11 y) Adolescents (12–18 y)	393 140 (35.6) 253 (64.4)
First-line treatment, *n* (%)	
Proton pump inhibitors	281 (71.5)
Swallowed topical corticosteroids	64 (16.3)
Dietary interventions	48 (12.2)
Age at diagnosis, years (mean ± SD)	11.1 ± 4.6
Diagnostic delay, years (mean ± SD) (*n* = 302)	1.9 ± 2.8
Male, *n* (%)	314 (79.9)
Year of diagnosis, *n* (%) (*n* = 388)	
< 2011	43 (11.1)
2011–2017	162 (41.7)
> 2017	183 (47.2)
Country of recruitment, *n* (%)	
Spain	329 (83.7)
Italy	60 (15.3)
Denmark	2 (0.5)
France	2 (0.5)
Type of facility, *n* (%)	
Pediatric	87 (22.1)
Adult	306 (77.9)
Treatment length until evaluation, days (median, IQR) (*n* = 239)	
Overall	98 (70–169)
For patients treated with proton pump inhibitors	94 (69–141)
For patients treated with swallowed topical corticosteroids	139 (97–654)
For patients treated with dietary options	114 (54–179)
EoE phenotype, *n*, % (*n* = 371)	
Inflammatory	334 (90.0)
Mixed	17 (4.6)
Stricturing	20 (5.4)
Endoscopic features of fibrosis at baseline, *n* (%) (*n* = 283)	92 (32.5)
Presence of rings, patients *n* (%)	80 (28.3)
Presence of stricture, patients *n* (%)	28 (9.9)

### Choice and effectiveness of first-line therapy

Among PPIs, omeprazole was the preferred drug (37%), used at 20 mg twice daily (43% of omeprazole treatments) (Suppl Table 2). As for STC, fluticasone propionate was more frequently prescribed than budesonide, with metered-dose formulation (inhalation or spray devices applied in the mouth and then swallowed), being the most common choice (34%) (Suppl Table 3). Finally, the vast majority of first-line dietary interventions were empiric elimination diets (77%) (Suppl Table 4).

Next, we evaluated and compared clinical and histological response rates to PPIs, STCs, and diets used in monotherapy (Table 2). No differences were found for clinical response overall, being 72%, 86%, and 73%, respectively (*p* = 0.178). Regarding histological endpoints, STC were the most effective choice, with 65% patients overall achieving < 15 eos/hpf, higher than the 43% and 42% obtained, respectively, for PPIs and diets (*p* = 0.017). Finally, and concordant with these results, clinico-histological response (clinical improvement together with < 15 eos/hpf) was better for STCs (66%) than for PPIs and diets (44% and 42%, respectively) (*p* = 0.018); with most patients (60%, 35%, and 28% of those respectively treated with SCT, PPIs, and diets) achieving clinico-histological remission (symptomatic improvement together with < 5 eos/hpf in esophageal biopsies).

**Table 2:** First-line therapies used according to demographical and clinical variables, and histological and clinical response rates for each treatment. EoE: eosinophilic esophagitis; eos/hpf: peak of eosinophils per high power field

	**Proton pump inhibitors,** ***n* (%)**	**Swallowed topical coritosteroids, *n* (%)**	**Dietary interventions, *n* (%)**	**Total, *n* (%)**	***p*-value**
**Clinical and histological response rates**					
Clinical improvement No response Not measured	184 (71.6) 73 (28.4) 24	49 (86) 8 (14) 7	32 (72.7) 12 (27.3) 4	265 (74) 93 (26) 35	0.178
Histological remission (< 5 eos/hpf) Histological response (5–14 eos/hpf) Eosinophil reduction No response Not assessed	84 (33.7) 22 (8.9) 17 (6.8) 126 (50.6) 32	29 (60.4) 2 (4.2) 3 (6.2) 14 (29.2) 16	13 (28.9) 6 (13.3) 2 (4.5) 24 (53.3) 3	126 (36.8) 30 (8.8) 22 (6.4) 164 (48) 51	**0.017**
Clinico-histological remission					
Yes No Not fully assessed	83 (34.7) 156 (65.3) 42	28 (59.6) 19 (40.4) 17	12 (27.9) 31 (72.1) 5	123 (37.4) 206 (62.6) 64	**0.001**
Clinico-histological response					
Yes No Not fully assessed	105 (43.9) 134 (56.1) 42	31 (66.0) 16 (34.0) 17	18 (41.9) 25 (58.1) 5	154 (46.8) 175 (53.2) 64	**0.018**
**Demographical and clinical variables**					
Sex, *n* (%) (*n* = 393)					
Male Female	222 (70.7) 59 (74.7)	51 (16.2) 13 (16.5)	41 (13.1) 7 (8.9)	314 (79.9) 79 (20.1)	0.591
Patients age, *n* (%) (*n* = 393)					
Children (3–11 years) Adolescents (12–18 years)	104 (74.3) 177 (70)	15 (10.7) 49 (19.4)	21 (15.0) 27 (10.7)	140 (35.6) 253 (64.4)	0.056
EoE phenotype, *n* (%) (*n* = 371)					
Inflammatory Mixed or stricturing	244 (73.1) 25 (67.6)	49 (14.7) 11 (29.7)	41 (12.3) 1 (2.7)	334 (90.0) 37 (10.0)	**0.023**
Endoscopic features of fibrosis, *n* (%) (n = 283)					
Absence Presence	157 (82.2) 63 (67.4)	12 (6.3) 20 (21.7)	22 (11.5) 10 (10.9)	191 (67.5) 92 (32.5)	**0.001**
Country of recruitment, *n* (%) (*n* = 389)					
Spain Italy	238 (72.3) 41 (68.3)	50 (15.2) 14 (23.3)	41 (12.5) 5 (8.3)	329 (84.6) 60 (15.4)	0.236
Recruiting center, *n* (%) (*n* = 393)					
Pediatric facility Adult facility	72 (82.8) 209 (68.3)	7 (8.0) 57 (18.6)	8 (9.2) 40 (13.1)	87 (22.1) 306 (77.9)	**0.024**

### Determinant factors involved in the selection of first-line therapy for pediatric EoE

Six variables were analyzed by univariate analysis to evaluate clinical and demographic factors determining first-line treatment choice: sex, age, EoE phenotype, features of fibrosis at baseline endoscopy, country, and recruiting facility (Table 2). STC treatments were preferably used in patients with a mixed/stricturing phenotype (*p* = 0.023), and when fibrotic features were found in baseline endoscopy (*p* = 0.001). Conversely, PPI prescriptions were more common in pediatric facilities compared to adult ones (*p* = 0.024). In addition, a trend towards more common use of dietary interventions in children under 12 years old, compared to adolescents, was also observed (*p* = 0.056).

A multinomial logistic regression model, adjusted by sex and type of facility (pediatric or adult), confirmed the relevance of these findings (Table 3). Being younger than 12 years old was associated with a preferred use of dietary therapy over drugs (PPI or STC) (*p* = 0.034). The presence of fibrotic features at baseline endoscopy and being recruited in Italy, rather than Spain, were independently associated with the use of STC over PPI or diet (*p* < 0.001 and *p* = 0.003, respectively).

**Table 3:** Multinomial logistic regression to identify variables associated with first-line treatment choice. CI: confidence interval; PPI: proton pump inhibitors; STC: swallowed topical corticosteroids; ORa: odds ratio adjusted by sex and attendance setting; ref: reference treatment

**Variable**	**Comparison**	**PPI**	**STC**		**Dietary interventions**	
			**ORa (95% CI)**	***p*-value**	**ORa (95% CI)**	***p*-value**
Age	Children *vs.* adolescent	Ref	1.48 (0.61–3.57)	0.570	2.43 (1.07–5.52)	**0.034**
Fibrotic features	Presence *vs*. absence	Ref	4.34 (1.91–9.84)	**< 0.001**	1.36 (0.58–3.18)	0.480
Country	Italy *vs*. Spain	Ref	3.91 (1.60–9.55)	**0.003**	1.18 (0.37–3.72)	0.781

### PPI treatment to induce clinico-histological remission and determinants for effectiveness

Given that PPI therapy was the preferred first-line option, and the highest number of patients belonged to this group, subsequent sub-analyses were performed to determine which variables could influence PPI effectiveness, measured as clinico-histological response. For this purpose, we identified 29 additional patients who received PPIs as second-line treatment, after failure to respond to STCs or dietary options. Of these, 23 were fully evaluated for clinical and histological responses, and they presented a lower response rate than when PPIs were used in first-line (30% *vs.* 44%), despite the difference not being significant (*p* = 0.211). Therefore, 262 patients with their clinico-histological response assessed were included in these analyses.

First, univariate statistical analyses were performed on variables affecting the PPI treatment itself (Table 4). The only variable associated with higher clinico-histological response was PPI dose, either as being high (double doses or higher) in the overall pediatric population (*p* = 0.030), or being higher than 1 mg/kg in those children under 12 years old (*p* = 0.041). Additional variables considered (type of PPI drug, intakes per day, days until evaluation, and line of treatment) did not significantly influence effectiveness. Next, similar analyses were undertaken on clinical and demographical variables (Table 5). Here, the type of recruiting facility was the only factor associated with the response to PPIs, as pediatric sites showed better response rates than adult ones (59% *vs.* 37%, *p* = 0.002).

**Table 4:** Univariate statistical analyses of the characteristics of proton-pump inhibitor (PPI) treatment that could determine effectiveness to induce clinico-histological response

	**No PPI-responder, *n* (%)**	**PPI-responder, *n* (%)**	**Total, *n* (%)**	***p*-value**
Type of PPI (*n* = 261)				
Esomeprazole	45 (49.5)	46 (50.5)	91 (34.8)	
Lansoprazole	43 (67.2)	21 (32.8)	64 (24.5)	
Omeprazole	50 (55.6)	40 (44.4)	90 (34.5)	0.186
Pantoprazole	7 (63.6)	4 (36.4)	11 (4.2)	
Rabeprazole	4 (80.0)	1 (20.0)	5 (2.0)	
Total	149 (57.1)	112 (42.9)	261	
Doses (*n* = 256)				
Low	30 (71.4)	12 (28.6)	42 (16.4)	**0.030**
High	114 (53.3)	100 (46.7)	214 (83.6)	
Total	144 (56.3)	112 (43.8)	256	
Doses (mg/kg) (for children under 12 years old) (*n* = 92)				
≤ 1.0 mg/kg	17 (81.0)	4 (19.0)	21 (22.8)	**0.041**
> 1.0 mg/kg	40 (56.3)	31 (43.7)	71 (77.2)	
Total	57 (62.0)	35 (38.0)	92	
Intakes/day (*n* = 253)				
1 time	20 (52.6)	18 (47.4)	38 (15.0)	0.600
2 times	123 (57.2)	92 (42.8)	215 (85.0)	
Total	143 (56.5)	110 (43.5)	253	
Days until evaluation (*n* = 161)				
43–70 days	20 (54.1)	17 (45.9)	37 (24.3)	
71–90 days	15 (57.7)	11 (42.3)	26 (17.1)	0.957
> 91 days	50 (56.2)	39 (43.8)	89 (58.6)	
Total	85 (55.9)	67 (44.1)	152	
Days until evaluation (median, IQR) (*n* = 161)	97 (74–168)	100 (70–140)	96 (70–145)	0.232
Line of treatment (*n* = 262)				
1st line	134 (56.1)	105 (43.9)	239 (91.2)	0.211
2nd line	16 (69.6)	7 (30.4)	23 (8.8)	
Total	150 (57.3)	112 (42.7)	262

**Table 5:** Univariate statistical analysis of those clinical and demographical variables that could determine the effectiveness of proton-pump inhibitor (PPI) treatment in inducing clinico-histological response

**Variable**	**No PPI-responder, *n* (%)**	**PPI-responder, *n* (%)**	**Total, *n* (%)**	***p*-value**
Sex (*n* = 262)				
Male	123 (58.6)	87 (41.4)	210 (80.2)	
Female	27 (51.9)	25 (48.1)	52 (19.8)	0.386
Total	150 (57.3)	112 (42.7)	262	
Country (*n* = 262)				
Spain	132 (58.9)	92 (41.1)	224 (85.5)	
Other	18 (47.4)	20 (52.6)	38 (14.5)	0.183
Total	150 (57.3)	112 (42.7)	262	
Age (*n* = 262)				
Children (< 12 years)	58 (62.4)	35 (37.6)	93 (35.5)	
Adolescent (> 12 years)	92 (54.4)	77 (45.6)	169 (64.5)	0.215
Total	150 (57.3)	112 (42.7)	262	
Age at diagnosis, years (mean ± SD)	12.8 ± 4.0	13.3 ± 3.7	13 ± 3.8	0.289
Phenotype (*n* = 258)				
Inflammatory	132 (55.7)	105 (44.3)	237 (91.9)	
Mixed or structuring	15 (71.4)	6 (28.6)	21 (8.1)	0.163
Total	147 (57.0)	111 (43.0)	258	
Presence of fibrosis at baseline (*n* = 210)				
No	83 (53.9)	71 (46.1)	154 (73.3)	
Yes	36 (64.3)	20 (35.7)	56 (26.7)	0.179
Total	119 (56.7)	91 (43.3)	210	
Recruiting center, *n* (%) (*n* = 262)				
Pediatric facility	28 (41.2)	40 (58.8)	68 (26.0)	**0.002**
Adult facility	122 (62.9)	72 (37.1)	194 (74.0)	
Total	150 (57.3)	112 (42.7)	262	

When these variables were included in a multivariate analysis, recruitment in a pediatric facility and the use of high PPI doses significantly associated with improved clinico-histological response to PPI therapy (*p* = 0.002 and *p* = 0.033, respectively) (Table 6). When analyses were adjusted by line of treatment and recruitment country, two more variables — age under 12 years old and having an inflammatory phenotype — showed a trend towards improved response (*p* = 0.051 and *p* = 0.085, respectively) (Table 6).

**Table 6:** Multivariate statistical analysis to identify variables that could determine the effectiveness of proton-pump inhibitor (PPI) treatment in inducing clinico-histological response. CI: confidence interval; OR: unadjusted OR; ORa: odds ratio adjusted by line of treatment and country

**Variable**	**OR (CI 95%)**	***p*-value**	**ORa (CI 95%)**	***p*-value**
Recruiting center (pediatric hospital vs. general hospital)	2.42 (1.38–4.26)	**0.002**	2.29 (1.22–4.30)	**0.010**
PPI dose (high vs. low)	2.19 (1.07–4.51)	**0.033**	2.32 (1.03–5.24)	**0.042**
Age (children vs. adolescent)	1.39 (0.83–2.33)	0.215	1.79 (0.99–3.21)	0.051
Phenotype (inflammatory vs. mixed or structuring)	1.99 (0.75–5.30)	0.170	2.46 (0.88–6.84)	0.085

### Effectiveness of induction PPI treatment to reverse fibrotic endoscopic features short term

From those patients treated with PPIs to induce EoE remission as first- or second-line therapy, 146 had data registered on EREFS score for both baseline and post PPI endoscopies, including 43% PPI-responders and 57% non-responders (Table 7). Reduction in total EREFS score from baseline was higher among responders (2.63 ± 1.56 *vs.* 0.40 ± 0.66; *p* < 0.001) compared to non-responders (2.94 ± 1.29 *vs.* 2.52 ± 1.53; *p* = 0.050).

**Table 7:** Changes in EREFS total score and subscores for each EREFS component according to clinico-histological response to proton-pump inhibitors (PPI). *p-values were calculated using McNemar test for qualitative variables and paired *t*-test for quantitative variables

	**PPI responder patients (*n* = 63)**			**PPI non-responder patients (*n* = 83)**		
	Baseline	After treatment	*p*-value^*^	Baseline	After treatment	*p*-value^*^
**EREFS (inflammatory features)**						
Edema, *n* (%)						
0	23 (36.5)	56 (88.9)	**< 0.001**	30 (36.1)	38 (45.8)	0.200
1	40 (63.5)	7 (11.1)		53 (63.9)	45 (54.2)	
Subscore (mean ± SD)	0.64 ± 0.48	0.11 ± 0.32	**< 0.001**	0.64 ± 0.48	0.54 ± 0.50	0.145
Furrows, *n* (%)						
0	15 (23.8)	51 (80.9)	**< 0.001**	19 (22.9)	26 (31.3)	0.248
1	48 (76.2)	12 (19.1)		64 (77.1)	57 (68.7)	
Subscore (mean ± SD)	0.76 ± 0.43	0.19 ± 0.39	**< 0.001**	0.77 ± 0.42	0.69 ± 0.47	0.179
Exudates, *n* (%)						
0	24 (38.1)	60 (95.2)	**0.001**	25 (30.1)	29 (34.9)	0.155
1	23 (36.5)	3 (4.8)		29 (34.9)	35 (42.2)	
2	16 (25.4)	0 (0)		29 (34.9)	19 (22.9)	
Subscore (mean ± SD)	0.87 ± 0.79	0.05 ± 0.22	**< 0.001**	1.05 ± 0.81	0.89 ± 0.78	0.068
Overall EREFS inflammatory subscore (mean ± SD)	2.31 ± 1.41	0.35 ± 0.63	**< 0.001**	2.49 ± 1.17	2.21 ± 1.38	0.061
**EREFS (fibrotic features)**						
Rings, *n* (%)						
0	51 (80.9)	60 (95.2)	**0.012**	64 (77.1)	68 (81.9)	0.607
1	8 (12.7)	3 (4.8)		12 (14.5)	10 (12.1)	
2–3	4 (6.4)	0 (0)		7 (8.4)	5 (6.0)	
Subscore (mean ± SD)	0.27 ± 0.63	0.05 ± 0.22	**0.005**	0.31 ± 0.62	0.24 ± 0.55	0.358
Strictures, *n* (%)						
0	60 (95.2)	63 (100)	-	79 (95.2)	77 (92.8)	0.500
1	3 (4.8)	0 (0)		4 (4.8)	6 (7.2)	
Subscore (mean ± SD)	0.05 ± 0.21	0	0.08	0.05 ± 0.21	0.07 ± 0.26	0.159
Overall EREFS fibrotic subscore (mean ± SD)	0.32 ± 0.75	0.05 ± 0.22	**0.007**	0.41 ± 0.73	0.34 ± 0.66	0.402
**EREFS (minor features)**						
Crepe paper esophagus, *n* (%)						
0	60 (95.2)	63 (100)	-	73 (87.9)	76 (91.6)	0.508
1	3 (4.8)	0 (0)		10 (12.1)	7 (8.4)	
Subscore (mean ± SD)	0.05 ± 0.21	0	0.08	0.12 ± 0.33	0.08 ± 0.28	0.320
**Total EREFS score (mean ± SD)**	2.63 ± 1.56	0.40 ± 0.66	**< 0.001**	2.94 ± 1.29	2.52 ± 1.53	0.050

When changes in EREFS inflammatory subscore components were analyzed, all the features (edema, furrows, and exudates) experienced a significant reduction from baseline (*p* < 0.001), while no significant changes were observed in non-responders. More importantly, EREFS fibrotic subscore for rings also decreased among responders (0.27 ± 0.63 *vs.* 0.05 ± 0.22, *p* = 0.005). Strictures were present at baseline in only three pediatric PPI-responder patients, and disappeared in all of them after PPI therapy. However, no changes for rings and strictures were noted among non-responders, and even an increase in the latter was found (Table 7).

## Discussion

This is the first analysis of the EoE CONNECT database focused on assessing treatment effectiveness exclusively in the pediatric population. To our knowledge, this is one of the largest real-world practice-based studies defining effectiveness of treatment in EoE in real life, and the first one to provide evidence on determinants for treatment choice, and on the effectiveness of PPIs in pediatric populations. According to our data, PPI monotherapy was the preferred first-line treatment choice in pediatric EoE patients, with an overall effectiveness similar to dietary options, but lower than STC. Treatment choice depended on patients’ age (with diets being more commonly used in younger children), endoscopic phenotypes at baseline (PPI being preferred in patients with inflammatory-predominant features at initial endoscopy), and country of patient’s origin (with PPIs being predominantly prescribed as a first-line choice in Spain). Effectiveness of PPI treatment was linked to the use of high doses and disease management by pediatric care facilities.

Our study reproduces results found in the literature previously [25], which shows that despite their limited effectiveness, PPIs are the main treatment option for EoE in both adults [19–21, 24] and children [20, 22, 23, 25]. Although there are more effective alternatives, clinicians seem to avoid pharmacological options based on STC in the initial therapeutic approach to EoE, and this trend is also reproduced among pediatricians. The effectiveness of PPIs in terms of clinical-histological remission in children, in general, parallels the figures reported in adults, although our results deserve further comment: as in previous reports, higher doses (both overall and adjusted by weight for age) achieved better results. However, we could not demonstrate that the number of doses into which the daily dose is divided was relevant. A recent study on 305 adults with EoE treated with PPI showed that doses of 20 mg of omeprazole per day, divided into two doses, were significantly superior to a single daily dose, with higher doses not achieving better results [36]. As previously shown in adults [19], the different PPI drugs used at equivalent doses provided comparable effectiveness. However, unlike what has been documented in adults, we were not able to demonstrate that prolonging treatment length beyond 8 weeks and up to 12 weeks associated with greater effectiveness [19], largely because few patients used short treatment times (the median time of PPI treatment until response assessment was 96 days).

Our work is the first, to our knowledge, that attempts to define reasons for the choice of first-line PPI therapy in younger EoE patients. Previous analyzes of the EoE CONNECT registry, on a predominantly adult population, had already shown significant differences in the choice of first-line therapies according to patients’ age, referral hospital, and EoE phenotype [37]. The present study shows that these factors also determined treatment choice in populations under 18 years old. The limited treatment strategies for EoE made it predictable that determinants would concur across different age groups in sites contributing to the same registry.

The other relevant result of this analysis is that we evaluated, for the first time, the ability of PPIs to reverse esophageal fibrosis features in children with EoE. Few patients showed rings or strictures at baseline endoscopy (only 32.5% of our series), thus coinciding with other reports in children [26], or were classified by their physicians as having a stricturing or mixed phenotype (10%), which was likely due to the short diagnostic delay in our cohort (only 2 years on average). However, the prevalence of both rings and strictures significantly reduced among PPI responders, while they showed no change among non-responders. These data confirm that PPIs also constitute an effective treatment for reversing the fibrosis associated with EoE, in the short term, in children. This had previously been demonstrated in a large series of adults [38], in whom endoscopic reversal of fibrosis was associated with an increase in esophageal caliber, measured by planimetry with endoFLIP [39] in a subgroup of cases.

The strengths of our study include the use of a large, multicenter registry of patients with EoE prospectively recruited at several European hospitals. All patients started EoE therapy at an age younger than 18-year-old, and data were prospectively collected for the majority. Treatment effectiveness was objectively evaluated in the vast majority of patients with homogeneous histological, clinical, and endoscopic criteria. However, some limitations should be also acknowledged, as our data came from somewhat expert facilities in the management of EoE and most patients were recruited at Spanish sites, and therefore not necessarily representative of practice patterns in other places. A reduced number of patients received STC or dietary interventions as first-line treatment for EoE, which may affect real effectiveness of these therapies. Furthermore, very few patients used combination therapies, and this prevented establishing their potential advantages over monotherapy. Anyway, no guidelines recommend such practice, and this is reflected in the expertise of recruiting sites in EoE CONNECT. The DSS score is not validated for measuring symptoms of EoE in children and adolescents, despite its demonstrated ability to capture changes induced by therapy [19, 37, 40, 41], even in randomized placebo-controlled trials [42]. Adherence to EoE treatment was not systematically assessed, and no efforts were made to determine medication compliance beyond the usual advice in each physician’s practice [27]; Therefore, the expected effectiveness of each intervention in a conventional clinical use setting is provided. Although we endeavored to thoroughly record and ensure data quality, a proportion of patients had missing data and bias in the information included cannot be ruled out. Finally, multivariate analysis was performed for the clinico-histological response endpoint, symptomatic improvement together with < 15 eos/hpf, as more strict criteria are not required for practice-based observational studies [43].

In conclusion, our results provide comparative effectiveness of first-line anti-inflammatory therapies in children and adolescents with EoE, and establish that the choice of initial therapy depends on the endoscopic features of the disease, patient’s age, and the country of origin of the patients. The effectiveness of PPI treatment was directly related to the use of high doses and management in pediatric facilities; among responders, this treatment option was able to significantly reverse the fibrotic findings associated with the disease. These data should be considered to inform future clinical practice guidelines and recommendations for the treatment of EoE in the pediatric age.

### Supplementary Information

Below is the link to the electronic supplementary material.

Supplementary file1 (DOC 55 KB)

## Data Availability

No datasets were generated or analysed during the current study.

## Abbreviations

CI: Confidence interval
DSS: Dysphagia Symptom Score
EoE: Eosinophilic esophagitis
IQR: Interquartile range
OR: Odds ratio
PPI: Proton pump inhibitors
STC: Swallowed topical corticosteroids
