# Supplementary material for: Determinant factors for first-line treatment choice and effectiveness in pediatric eosinophilic esophagitis: an analysis of the EUREOS EoE CONNECT registry
Source: Eur J Pediatr. 2024 May 31;183(8):3567–78. doi: 10.1007/s00431-024-05618-z (PMC11263422; doi:10.1007/s00431-024-05618-z)
Supplement: Supplementary file 1 — Supplementary file1 (DOC 55 KB) [file 431_2024_5618_MOESM1_ESM.doc]

**Supplementary Table 1.** First-line treatment according to type of therapy.

|  | **Patients** | |
| --- | --- | --- |
|  | **n** | **%** |
| Proton pump inhibitors | 281 | 67.5 |
| Swallowed topical steroids | 64 | 15.4 |
| Dietary interventions | 48 | 11.5 |
| Proton pump inhibitors + swallowed topical steroids | 15 | 3.6 |
| Proton pump inhibitors + dietary interventions | 6 | 1.4 |
| Endoscopic dilatation | 1 | 0.3 |
| Proton pump inhibitors + endoscopic dilation | 1 | 0.3 |
| **Total** | **416** | **100** |

**Supplementary Table 2.** Type, dose and frequency of proton-pump inhibitors used in monotherapy as first-line treatment.

|  | **Patients** | |
| --- | --- | --- |
|  | **n** | **%** |
| **Omeprazole**  40 mg twice daily  40 mg once daily  20 mg twice daily  20 mg once daily  10 mg twice daily  2 mg/Kg once day | **103**  27  11  40  9  4  2 | **36.6**  29.0  11.8  43.0  9.7  4.3  2.1 |
| **Esomeprazole**  40 mg twice daily  40 mg once daily  20 mg twice daily  20 mg once daily  10 mg twice daily | **91**  45  5  14  4  3 | **32.4**  63.4  7.0  19.7  5.6  4.2 |
| **Lansoprazole**  30 mg twice daily  30 mg once daily  15 mg twice daily  15 mg once daily | **69**  36  7  14  2 | **24.6**  61.0  11.9  23.7  3.4 |
| **Pantoprazole**  40 mg twice daily  20 mg twice daily | **12**  6  2 | **4.3**  75.0  25.0 |
| **Rabeprazole**  40 mg twice daily  20 mg twice daily | **5**  2  2 | **1.8**  50.0  50.0 |
| **Unknown** | **1** | **0.3** |
| **Total** | **281** | **100** |

**Supplementary Table 3.** Type, formulation, dose and frequency of swallowed topical corticosteroids used in monotherapy as first-line treatment.

|  | **Patients** | |
| --- | --- | --- |
|  | **n** | **%** |
| Fluticasone propionate nasal drop suspension swallowed instead of applied inside the nose  400 µg twice daily  400 µg once daily  200 µg twice daily  Unknown | **17**  10  3  3  1 | **26.6**  58.8  17.6  17.6  5.8 |
| Fluticasone propionate metered-dose (inhalation or spray devices) applied in the mouth and then swallowed  1000 µg twice daily  500 µg twice daily  250 µg four times daily  250 µg thrice daily  250 µg twice daily  250 µg once daily  Unknown | **22**  1  5  4  2  8  1  1 | **34.3**  4.5  22.7  18.2  9.1  36.5  4.5  4.5 |
| Budesonide, oral viscous prepared by a pharmacist  1 mg twice daily  1 mg once daily  0.5 mg twice daily  0.5 mg once daily | **13**  8  2  2  1 | **20.3**  61.5  15.4  15.4  7.7 |
| Budesonide, oral disintegrating tablet  1 mg twice daily  1 mg once daily | **3**  1  2 | **4.7**  33.3  66.7 |
| Budesonide, oral viscous prepared by a pharma company  Unknown | **3**  3 | **4.7**  100 |
| Budesonide, metered-dose inhaled or spray  1 mg twice daily  1 mg once daily | **3**  2  1 | **4.7**  66.7  33.3 |
| Budesonide, home-made oral viscous  1 mg twice daily  1 mg once daily | **3**  2  1 | **4.7**  66.7  33.3 |
| **Total** | **64** | **100** |

**Supplementary Table 4.** Type of dietary interventions used in monotherapy as first-line treatment.

|  | **Patients** | |
| --- | --- | --- |
|  | **n** | **%** |
| **Empiric food elimination diets**  Six-food  Four-food  Two-food  Single-food | **37**  13  1  12  11 | **77.1**  35.1  2.7  34.3  29.7 |
| **Allergy testing-driven food elimination** | **8** | **16.7** |
| **Other types of diets** | **3** | **6.2** |
| **Total** | **48** | **100** |
